# An Outbreak of *Salmonella* Typhimurium Infections Linked to Ready-To-Eat Tofu in Multiple Health Districts — Ontario, Canada, May–July 2021

**DOI:** 10.15585/mmwr.mm7232a1

**Published:** 2023-08-11

**Authors:** Victoria Osasah, Yvonne Whitfield, Janica Adams, Affan Danish, Richard Mather, Mehdi Aloosh

**Affiliations:** ^1^Public Health Ontario, Toronto, Ontario, Canada; ^2^Johns Hopkins University Bloomberg School of Public Health, Baltimore, Maryland; ^3^Department of Health Research Methods, Evidence and Impact, McMaster University, Hamilton, Ontario, Canada.

**Summary**

**What is already known about this topic?**

*Salmonella* Typhimurium is a serovar commonly implicated in foodborne illnesses linked to animal product consumption.

**What is added by this report?**

During May–July 2021, an outbreak of *S.* Typhimurium involving 38 cases in 10 public health districts in Ontario, Canada was linked to consumption of tofu, suggesting a novel outbreak-associated *S.* Typhimurium food vehicle. Lapses in sanitation and recommended heat processing likely resulted in product contamination.

**What are the implications?**

Tofu has not been previously linked to nontyphoidal *Salmonella* outbreaks. Public health communications to consumers and food establishments should aim to increase awareness of the possible transmission of *Salmonella* through ready-to-eat soy products. In addition, interventions need to target production and all parts of the supply chain, with additional safeguarding steps that minimize growth of *Salmonella* in soy-based products.

## Abstract

From May to mid-August 2021, the Ontario, Canada provincial public health agency, Public Health Ontario, in collaboration with local public health authorities and federal food safety partners, investigated a spatiotemporal cluster of 38 patients with *Salmonella* Typhimurium infections across multiple public health districts in Ontario. Five (13%) patients were hospitalized; no deaths were reported. The outbreak was linked to consumption of ready-to-eat seasoned tofu from one manufacturer that was distributed to multiple Ontario restaurants. Isolates from the seasoned tofu were within one or fewer allele differences to the outbreak strain by whole genome sequencing. Evidence from food safety investigations conducted by local public health authorities and the Canadian Food Inspection Agency (CFIA) revealed that unsanitary conditions could have led to cross-contamination of the tofu, and insufficient heating of the tofu at the production level likely resulted in failure to eliminate the pathogen. The CFIA issued a food recall for the tofu at hotel, restaurant, and institution levels. Tofu was identified as a novel outbreak-associated food vehicle for *S.* Typhimurium in this outbreak. Interventions that target the production level and all parts of the supply chain and include additional safeguarding steps that minimize microbial growth are important.

## Epidemiologic Investigation and Findings

On July 5, 2021, Public Health Ontario (PHO) identified, via routine surveillance, three cases of *S.* Typhimurium infections across multiple public health districts (known as public health units) in Ontario, with four or fewer allele differences in isolates by whole genome multilocus sequence typing (wgMLST), suggesting a common exposure source. By July 9, six more cases were reported to PHO. In collaboration with local, provincial, and federal health authorities, PHO initiated an outbreak investigation. Cases continued to be reported across Ontario through mid-August; among 10 public health districts, incidence ranged from ≤0.2 to 2.9 cases per 100,000 persons. Although *S.* Typhimurium is one of the most common serovars in Ontario, the outbreak strain was not related to any existing clusters or isolates in PulseNet Canada, a national surveillance system that collects information on foodborne-related illnesses caused by specific pathogens. This activity did not require ethics approval because the operations were within the purview of PHO’s legislated mandate.*

PHO defined a confirmed case as an infection with *S.* Typhimurium in a resident of or a visitor to Ontario occurring after April 30, 2021, with a genomic sequence pattern consistent with (≤10 wgMLST allele differences) the outbreak strain. Thirty-eight cases were reported across 10 of 34 public health districts in Ontario. Symptom onset dates ranged from May 16 to July 31, 2021. The median patient age was 27 years (range = 1–87 years); 25 (66%) patients were aged ≥24 years, and 21 (55%) identified as female. Five (13%) patients were hospitalized, and no deaths were reported.

Patients with laboratory-confirmed *Salmonella* infections related to the whole genome sequencing (WGS) cluster were interviewed by local and provincial public health investigators in the 10 affected Ontario public health districts. Using standardized hypothesis-generating questionnaires, investigators recorded food exposure and other risk factors associated with animal and occupational exposure during the 7-day period preceding symptom onset. Information on restaurants and shops visited during the exposure period was collected to further identify any common food locations reported among the patients.

The proportions of reported risk factors were compared with corresponding reference values from the Foodbook report, a population-based telephone survey conducted in all Canadian provinces within a 1-year period during 2014–2015 that focused on describing foods eaten by Canadians during a 7-day period, to guide outbreak investigations and responses (*1*). An exact probability test was applied to measure the statistical significance of the consumption rates of patients with outbreak-confirmed illness when compared with the Foodbook reference values. Differences with associated p-values <0.05 were considered statistically significant.

Illness onset dates clustered from late June through mid-July (Figure), suggesting an ongoing common-source exposure. Thirty patients were interviewed (response rate = 79%), and 19 (63%) reported being on a vegetarian or vegan diet. Among the 25 patients who provided a response for “consumption of tofu,” 19 (76%) responded that they had consumed or probably consumed tofu, representing a significantly higher proportion than the proportion of the general population surveyed in the Foodbook report who reported eating tofu (3%; p<0.001). Other food items reported by patients that were statistically significantly more likely to be consumed were explored (such as non-dairy milk, vegetables, nuts, and avocado), but they lacked specificity by product type, brand name, and place of purchase. Among the 19 patients who reported consuming tofu, 16 purchased seasoned tofu either at one of 11 restaurant franchise locations or one of three nonfranchise restaurant locations across Ontario, before their illness onset.

**FIGURE:**
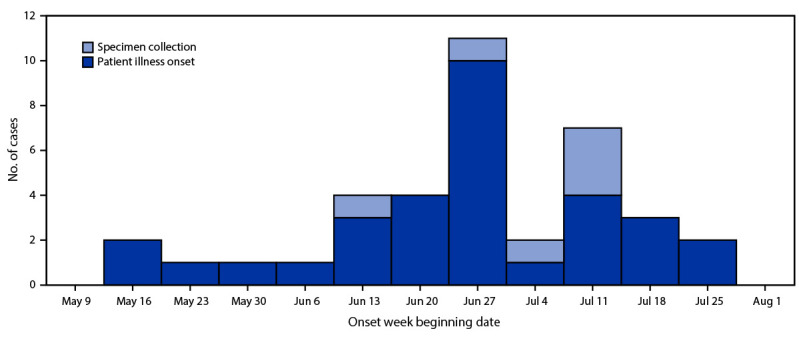
Week of illness onset and specimen collection (N = 6) for patients infected with a *Salmonella* Typhimurium outbreak strain (N = 32) — Ontario, Canada, May–August 2021

## Food Safety and Laboratory Investigation and Findings

All nonclinical specimens and isolates from clinical specimens were submitted to Public Health Ontario’s laboratory (PHOL), a clinical and environmental reference laboratory in Ontario, for analysis. Isolates from all outbreak-confirmed cases underwent WGS at PHOL and the Public Health Agency of Canada’s National Microbiology Laboratory. Isolates with four or fewer wgMLST allele differences were considered related by WGS. During the outbreak investigation, an isolate from a case in Quebec closely related by WGS to the outbreak strain was identified in PulseNet Canada.

As a result of the epidemiologic evidence, local investigators and Canadian Food Inspection Agency (CFIA) authorities conducted investigations at restaurants where patients reported consuming seasoned tofu during the 7-day period before symptom onset. Additional investigations were conducted once a common manufacturer was identified. A total of 16 opened and closed specimens of the seasoned tofu product were collected from 10 restaurants and the manufacturer. After extensive food safety investigations, *S.* Typhimurium was isolated from three open specimens of seasoned tofu obtained from one of the restaurant franchise locations; the sequenced isolates were closely related by WGS to those from outbreak-confirmed cases. *Salmonella* was not detected in other food specimens produced by the manufacturer.

Food safety investigations revealed that seasoned tofu from the same manufacturer was served across all 14 restaurants. The tofu was identified as a ready-to-eat food product that was produced by a manufacturer in Ontario and commercially sold in 250-g (8.8-oz) and 500-g (17.6-oz) packages. Restaurants purchased the product as a 500-g vacuum-sealed package.

Food safety investigations identified the absence of a heat treatment process after the addition of seasoning to the packaged 500-g product, which was also sold online to other provinces including Quebec; the 250-g packaged product did undergo additional heat treatment. No illnesses were linked to the 250-g packaged product. Several infractions were observed at the manufacturing plant, including poor sanitation of the processing equipment and the absence of a food safety plan or a food sampling program.

## Public Health Response

CFIA issued a food recall for the 500-g tofu product. Local public health inspectors ensured that existing products were removed from distribution and destroyed across implicated restaurants and the manufacturing plant. As a corrective action within the manufacturing facility, a heat treatment step after the addition of the seasoning before packaging was applied.

## Discussion

Tofu was identified as the source of an outbreak of *S.* Typhimurium in Ontario in 2021. Investigators hypothesized that unsanitary conditions at the production facility could have led to contamination of the tofu after production and before packaging, but the absence of an additional heating step during production likely resulted in failure to eliminate the pathogen. Tofu is a novel outbreak-associated food vehicle for this pathogen and has not been implicated in previous outbreaks. Soy products, including tofu, are uncommon vehicles for foodborne illnesses. Among previously published outbreaks linked to soy products, only one outbreak involved *Salmonella* (*Salmonella enterica* paratyphi) (*2*). Although tofu has been implicated in outbreaks associated with other pathogens, there are no published reports of tofu-associated nontyphoidal *Salmonella* outbreaks (*3*,*4*); however, the growth or presence of *S.* Typhimurium on soy products has been detected in microbiological food studies (*5*,*6*).

Novel outbreak-associated food vehicles can emerge because of evolution of a pathogen or a change in dietary trends (*7*). This outbreak largely affected patients who had adopted a vegan or vegetarian diet. An estimated 5% of Canadians adhere to a plant-based diet (*8*). In addition, age and gender differences are apparent among persons adhering to plant-based diets such as vegetarianism, which is practiced more commonly among females and younger adults (*9*), consistent with the patient demographics in this outbreak.

The implication of detecting *S.* Typhimurium in tofu as a novel outbreak-associated food vehicle is of public health importance because of the global increase in the consumption of plant-based proteins and the associated high disability-adjusted life years associated with *S.* typhimurium infection^†^ (*10*). Improved guidance regarding the processing and handling of plant-based proteins in the supply chain is warranted to eliminate the growth and transmission of foodborne disease pathogens.

## Footnotes

* Ontario Agency for Health Protection and Promotion Act, SO 2007, c 10, Sch K.

†
https://www.bloomberg.com/company/press/plant-based-foods-market-to-hit-162-billion-in-next-decade-projects-bloomberg-intelligence/
